# Biofortified Cassava with Pro-Vitamin A Is Sensory and Culturally Acceptable for Consumption by Primary School Children in Kenya

**DOI:** 10.1371/journal.pone.0073433

**Published:** 2013-08-30

**Authors:** Elise F. Talsma, Alida Melse-Boonstra, Brenda P. H. de Kok, Gloria N. K. Mbera, Alice M. Mwangi, Inge D. Brouwer

**Affiliations:** 1 Division of Human Nutrition, Wageningen University, Wageningen, The Netherlands; 2 Applied Nutrition Program, Department of Food Technology and Nutrition, University of Nairobi, Nairobi, Kenya; Vanderbilt University, United States of America

## Abstract

**Background:**

Biofortification of cassava with pro-vitamin A can potentially reduce vitamin A deficiency in low-income countries. However, little is known about consumer acceptance of this deep yellow variety of cassava compared to the commonly available white varieties. We aimed to determine the sensory and cultural acceptability of the consumption of pro-vitamin A rich cassava in order to identify key factors predicting the intention to consume pro-vitamin A rich cassava by families with school-aged children in Eastern Kenya.

**Methods:**

Sensory acceptability was measured by replicated discrimination tests and paired preference tests among 30 children (7–12 yr) and 30 caretakers (18–45 yr) in three primary schools. Cultural acceptability was assessed with a questionnaire based on the combined model of The Theory of Planned Behavior and The Health Belief Model in one primary school among 140 caretakers of children aged 6 to 12 years. Correlations and multivariate analyses were used to determine associations between summed scores for model constructs.

**Results:**

Caretakers and children perceived a significant difference in taste between white and pro-vitamin A rich cassava. Both preferred pro-vitamin A rich cassava over white cassava because of its soft texture, sweet taste and attractive color. Knowledge about pro-vitamin A rich cassava and it's relation to health (‘Knowledge’ ((β = 0.29, P = <.01)) was a strong predictor of ‘Health behavior identity’. Worries related to bitter taste and color (‘Perceived barriers 1’ (β = −0.21, P = .02)), the belief of the caretaker about having control to prepare cassava (‘Control beliefs’ (β = 0.18, P = .02)) and activities like information sessions about pro-vitamin A rich cassava and recommendations from health workers (‘Cues to action’(β = 0.51, P = <.01)) were the best predictors of intention to consume pro-vitamin A rich cassava.

**Conclusions:**

Pro-vitamin A rich cassava is well accepted by school children in our study population.

## Introduction

Biofortification of crops can increase the micronutrient content using traditional breeding methods or modern biotechnology. Biofortification of staple foods with micronutrients is regarded as a sustainable approach to reduce micronutrient malnutrition. It could potentially benefit people in rural and remote areas with limited access to alternative possible interventions such as supplementation or introducing fortified food products [1], [2], [3].

Vitamin A deficiency (VAD) is a public health problem among young children and pregnant women in low-income countries. Vitamin A is an essential micronutrient for maintaining eye sight, immune function, and growth and development. VAD is caused by low dietary intake of vitamin A in combination with malabsorption and high excretion of vitamin A due to common illnesses [4]. In Kenya, the VAD prevalence is classified as severe by WHO with more than 60% of preschool children having moderate or severe VAD [5].

Cassava (*Manihot esculenta Crantz*) is a starchy staple food that is consumed widely in tropical and subtropical Africa, Asia and Latin America. This drought resistant crop can grow on poor soils with little water and can be harvested when needed, providing households with an alternative when the harvest of other crops fails [6]. Cassava cultivars that are naturally rich in pro-vitamin A were identified by the International Centre for Tropical Agriculture in Columbia (CIAT). New cultivars with a total carotenoid content range of 100–10,000 µg/100 g in fresh cassava have been developed by selective breeding of cassava with high-carotene germplasm by CIAT and the International Institute of Tropical Agriculture (IITA) in Nigeria [7]. These cultivars were introduced in Kenya for an efficacy trial conducted in 2012 among school aged children [8].

Biofortification changes the color of cassava roots from white to deep yellow, due to the increase in pro-vitamin A content. Not only appearance but also taste can be influenced due to lower dry matter concentration associated with higher pro-vitamin A concentration [7]. For biofortification programs to be successful, the biofortified crop needs to be accepted by both farmers and consumers [9]. Consumer acceptance depends on the sensory characteristics and beliefs and practices in the community [2]. Little is known about consumer acceptance of these new cultivars of cassava.

The Theory of Planned Behavior (TPB) and the Health Belief Model (HBM) are two psychosocial theories that are widely used in explaining food-related behaviors. According to the TPB, behavior is a conscious effort mediated by intention [10] and in the HBM health behavior is expected to result from a set of core beliefs [11]. A combination of these two theories has been shown to improve the ability to explain nutrition behavior [12], [13], [14]. Most acceptability studies in developing countries are entirely qualitative and use for example focus group discussions and interview methods in study populations selected by convenience [15], [16], without verifying the associations between beliefs or attitudes and consumption in larger representative populations. As such, a lot more insight can be gained into possible ways to influence nutrition behavior. Willingness to pay studies [17], [18] are also used to assess acceptability of new foods, though not useful in our situation as our population depends mainly on their own production of crops.

In this study, we aimed to determine the sensory and cultural acceptability of the consumption of pro-vitamin A rich cassava in order to identify key factors predicting the intention to consume pro-vitamin A rich cassava by families with school-aged children.

## Materials and Methods

### Ethic statement

Research authorization was given by the Ministry of Higher Education, Science and Technology, Kenya. The study was exempted from ethical approval by the Ethics and Research Committee of Kenyatta National Hospital in Kenya, because the study used non-invasive methods and because it was part of a larger study that had approval (P2293/07/2011). The study was explained to and written informed consent was obtained from the caretakers on behalf of their children before the study started.

### Study area

The research was conducted in Kibwezi district of Eastern Province in Kenya, an arid to semi arid area (ASAL) where cassava is grown but not consumed daily. This research site was chosen based on eligibility for conducting an efficacy study with pro-vitamin A rich cassava among primary school children at a later point in time.

### Study participants and sampling

The study comprised two parts: 1) a sensory evaluation including a difference test and a preference test [19], [20], and 2) a cultural acceptability study using a questionnaire. Three public primary schools were chosen out of 15 eligible schools in the area, based on high prevalence of vitamin A deficiency measured in the previous year (unpublished results). For the sensory evaluation, 10 caretakers with a child between 6 and 12 years old were recruited in each of the three schools. For the cultural acceptability study, 140 caretakers participated in only one out of the three schools, due to exposure of the other two schools to information about pro-vitamin A rich cassava the previous year. All caretakers of children in the age of 6 to 12 years were eligible and were only interviewed for one randomly chosen child if they had more than one eligible child. Any other eligible siblings were excluded, as dietary habits tend to be the same within one household. Data was collected over a period of four weeks in May 2011 by three trained enumerators for the sensory study and by six trained enumerators for the cultural acceptability study.

### Study measurements

For the sensory study a pro-vitamin A rich cassava variety (*97/1170*, cultivated by traditional breeding techniques) and a commonly available white cassava variety (*Ex-Mariakani*) were peeled and cooked for 30 minutes in water and manually mashed with a little oil and salt. Participants received instructions on the tests and a demonstration was given with mango and pineapple. The difference (or triangle) test consisted of six rounds per subject, each with three samples, of which one was different. Each round contained a randomly assigned, different serving order. Participants were blindfolded prior to serving the samples to prevent the color of the cassava from influencing the decision. The participant tasted the three samples and indicated to the enumerator which sample was the odd one. They were allowed to swallow the sample but had to rinse their mouths with water after each taste. This test was based on the alternative hypothesis that the probability of the participant making the correct decision when perceiving a difference between samples had to be larger than one out of three (i.e. H_a_:P_t_ >1/3) [19]). The preference test was done after the difference tests with a randomly assigned serving order of the two cassava varieties. Participants were not blindfolded anymore, and were asked to taste the two samples, to choose the variety they preferred the most and to write down or mention the reason to the enumerator. They were allowed to swallow the sample but had to rinse their mouths with water after each taste. We hypothesized that participants would have a preference for one type of cassava (H_a_: P (A) ≠ P(B)) [20].

**Figure 1:**
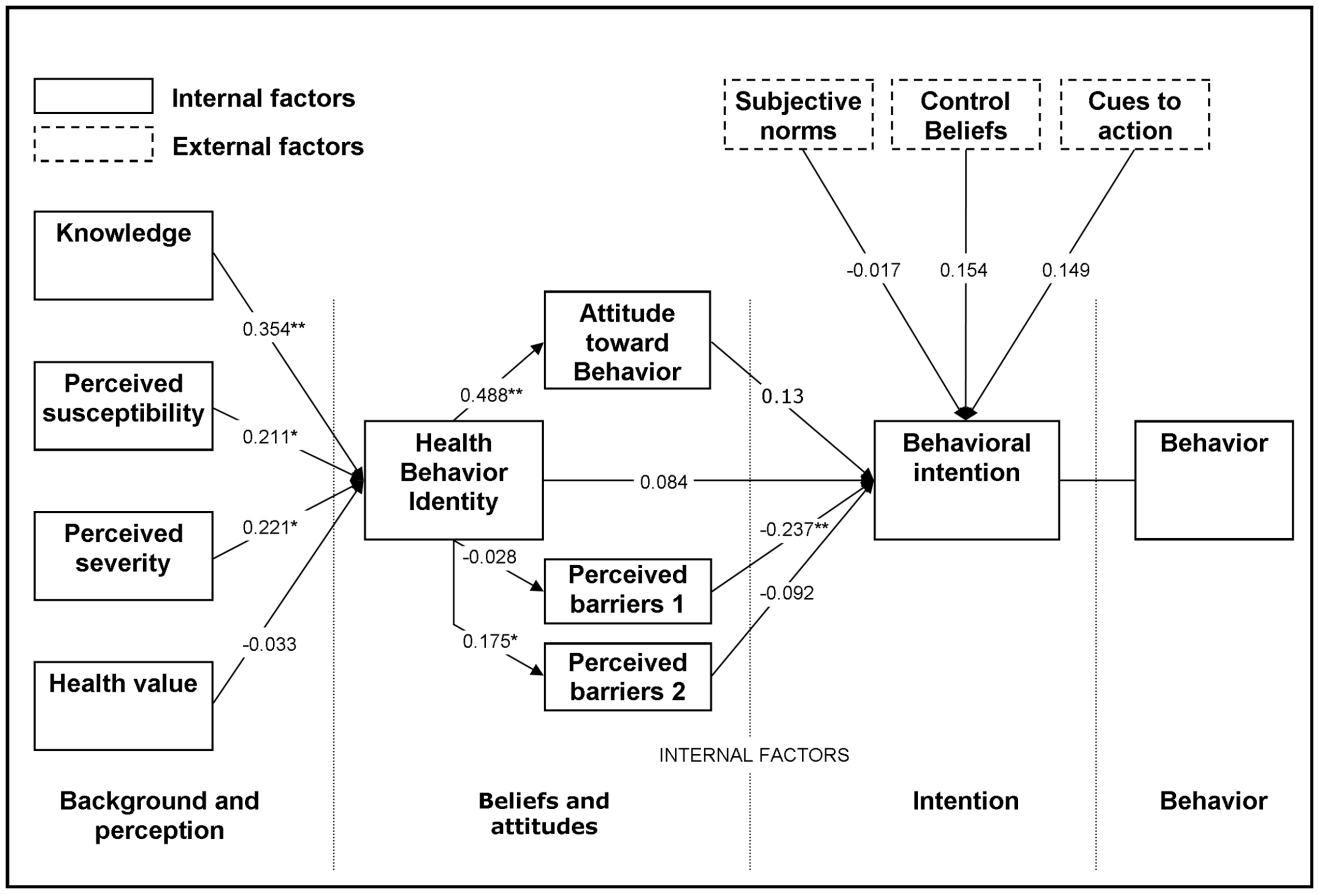
Correlations of the constructs using the combined health belief and theory of planned behavior models. (Adjusted model based on Sun et al 2006) *P<0.05, ** P<0.001 (both two tailed) The model: The model is based on the idea that the construct Behavioral intention (intention to feed child with pro-vitamin A rich cassava) is an important predictor for Behavior (feeding the child pro-vitamin A rich cassava) The constructs related to ‘Background and perception’, are ‘Knowledge’ (about pro-vitamin A rich cassava and VAD), Perceived susceptibility' (perception of developing VAD), ‘Perceived severity’ (notion of seriousness of developing VAD), and ‘Health value’ (notion of priority to stay healthy). The constructs related to ‘Beliefs and attitudes’, are ‘Health behavior identity’ (notion that it is good to eat vitamin A pro-vitamin A rich cassava), ‘Perceived barriers’ (perceived obstacles which prevent the consumer from eating pro-vitamin A rich cassava) and ‘Attitude towards behavior’ (positive or negative feeling towards eating pro-vitamin A rich cassava). The external factors consist of the constructs ‘Subjective norms‘ (perceived social pressure to consume pro-vitamin A rich cassava), ‘Control beliefs (perceived ability to consume pro-vitamin A rich cassava), and ‘Cues to action’ (external triggers, which stimulate to consume pro-vitamin A rich cassava).

Cultural acceptability was assessed by a questionnaire based interview. The interview was conducted with caretakers of the children as children below 12 years of age may not provide reliable answers and are unable to handle scale scores [21]. The questionnaire consisted of two parts; the first part aimed to collect information on general socio-demographic characteristics followed by a second part comprising statements related to the constructs of the combined TPB and HBM model (see Figure 1). For the latter, 67 statements were formulated based on findings in literature and a preparatory food ethnographic study on cassava and vitamin A deficiency, including a pile sort, a food attributes and differences study, and focus group discussions among caretakers and key-informants [22] [23]. The statements were sorted into 12 constructs according to the model. Example statements per constructs are given in Table 1 and an explanation of the constructs is given in the footnote of Figure 1. Respondents were asked to indicate their level of agreement with a statement on a 5-point Likert scale, ranging from strongly disagree to strongly agree. Exceptions were made for the constructs ‘Behavior’ and ‘Behavioral Intention’ for which a time scale was used: never, once or less per month, 2–3 times per month, once a week, two or more times a week. The constructs ‘Knowledge’ and ‘Perceived Severity’ were measured with a 5-point Likert scale with an additional column ‘don’t know'. Statements of all constructs were scored from 1 to 5 with the item ‘don’t know' being scored as neutral. However the constructs ‘Behavior’ and ‘Behavioral intention’ were scored 0 to 4. Questions within the constructs ‘Attitudes towards behavior’ and ‘Subjective norms’ consisted of a statement and an evaluation of that statement. Statements were scored 1 to 5 and evaluations were scored −2 to 2 and the two outcomes were multiplied, resulting in a score possibility of −10 to 10 for that statement. Questions for unmarried women within the construct ‘Subjective norms’ were recorded as not applicable and scored as neutral. The questionnaire was translated into Kiswahili and correctness was checked by back translating into English. The questionnaire was pretested, which resulted in small adjustments in the interpretation and explanation of the questions. After the interview the statements were scored and all scores within a construct were summed, resulting in a total construct score per respondent.

**Table 1:** Internal consistency and median scores of the constructs (n = 140).

Construct	Number of items	Cronbach α	Median	Range of score*	Example statements
Knowledge	5	0.61	19	13–25	Vitamin A can prevent infections
Perceived susceptibility	2	0.69	10	2–10	School children are at risk of developing VAD
Perceived severity	6	0.80	26	10–30	Lack of vitamin A makes my child susceptible to diseases
Health value	2	0.56	10	4–10	That my child can properly see during dusk or dawn is the most important thing in my life
Health behavior Identity	1	N/A	3	1–5	Eating pro-vitamin A rich cassava is good for my child
Attitudes towards behavior	9	0.73	57	−13–80	a) Pro-vitamin a rich cassava is nutritious. b) I find it important to prepare food that is nutritious for my child
Perceived barriers – 1	4	0.96	13	4–20	I worry about the yellow color of pro-vitamin A rich cassava
Perceived barriers – 2	10	0.60	40	16–50	Cassava is expensive to buy
Subjective norms	5	0.66	21	−20–50	a) My husband determines when to prepare cassava. b) The opinion of my husband on what to prepare is important to me
Control beliefs	3	0.72	15	9–15	I decide what food to buy for my household
Cues to action	8	0.80	36	11–40	School children in my household suffering from VAD would make me decide to buy pro-vitamin A rich cassava
Behavioral intention	1	N/A	4	0–4	Will you prepare pro-vitamin A rich cassava for your child in the future?

* Range refers to minimum and maximum score for each construct in the study.

### Statistical Analyses

Data were analyzed with the Statistical Package for Social Science (IBM SPSS statistics 19.0). All statistical tests were 2-tailed and p values <0.05 were considered statistically significant.

In the sensory evaluation, the responses for the difference test were not independent and probability of between and within person variance existed. This variance is known as over dispersion and is measured by gamma (γ), a value between 0 and 1. When γ is significantly greater than 0, the beta-binomial model has to be used to avoid an underestimation of the standard error. When γ is 0, there is no over dispersion and the binomial model can be used. To verify that γ was significantly greater than 0, Tarone's Z statistics [24] was calculated to test the goodness of fit of the binomial distribution against the beta-binomial distribution. When Tarone's Z statistics is greater than 1.645 the beta-binominal distribution has a better fit (α = 0.05, one tailed). To identify the number of correct responses needed for a significant difference in taste the critical values by Lawless and Heymann [19] were used for the binomial model, and those by Bi and Ennis [25] for the beta-binomial model. For the preference test, only one answer was given per respondent and therefore the critical values by Lawless and Heymann [20] were used to evaluate whether the minimal values for statistical significance were reached.

For the cultural acceptability evaluation, descriptive statistics were used to examine socioeconomic characteristics of the caretakers and to calculate median scores of the constructs. Multiple item constructs were tested for reliability and internal consistency using Cronbach α and item-total correlation [26]. Consistency within a construct is achieved when the Cronbach α is 0.7 or higher and when the corrected item-total correlation of all items in a construct is higher than 0.30. If exclusion of an item resulted in a higher Cronbach α value for the total item set, the item was excluded from the construct to enhance the consistency of the construct. The consistency within the construct ‘Perceived barriers’ was low (Cronbach α <0.5). We separated the initial 16 statements into two distinct constructs: ‘Perceived barriers 1’ reflecting the worries about taste and appearance of pro-vitamin A rich cassava (4 statements) and ‘Perceived barriers 2’ representing the believed facts about pro-vitamin A rich cassava (12 statements). Within construct ‘Perceived barriers 2‘ two statements were excluded to improve consistency. In total, 11 statements out of 67 were deleted from the initial questionnaire to improve reliability. Final Cronbach α values ranged from 0.56 to 0.80 which indicates medium to sufficient reliability of the constructs (Table 1). Median scores of the constructs were high in comparison to the range of scores which showed that caretakers tended to agree with the statements.

Spearman correlation was determined to assess bivariate associations between constructs. Multiple regression analyses was used to determine which constructs significantly predict the intention to consume pro-vitamin A rich cassava. All models were adjusted for interviewer, age and education of the caretaker.

## Results

In the sensory study a total of 30 caretakers (97% female), 31.6±7.1 (mean ± SD) years old, and 30 children (63% female), 8.9±1.6 years old, participated independently. Respondent variability for the discrimination test was moderate for the adults group (y = 0.17) and 0 for children (y = −0.01). Tarone's Z statistic was 3.6 for caretakers and −0.2 for children. Therefore the beta-binominal model was used for caretakers and the binomial model for children in the discrimination test. Both caretakers and children were able to detect a significant difference between pro-vitamin A rich and white cassava: 180 difference tests were administered in both groups and 130 correct answers were observed for the caretakers and 89 for the children (Table 2). Both caretakers and children preferred the pro-vitamin A rich cassava over the white cassava. Out of the 30 answers in both groups, 22 caretakers and 21 children had a preference for pro-vitamin A rich cassava (Table 3). Characteristics attributed to pro-vitamin A cassava were: attractive color, soft texture and sweet taste.

**Table 2:** Results for the difference test with pro-vitamin A rich and white cassava.

Difference test	Caretakers (n = 30)	Children (n = 30)
Participant variability (y)	0.17*	0.00
Appropriate model	Beta-binomial	Binomial
Max. no. of correct responses	180	180
No. of correct responses needed for significance	75	71
No. of correct responses observed	130**	89**
Discrimination µ Test: µ_0_ = 1/3	0.72	0.49

* =  significant (α = 0.05) using Tarone's Z statistic.

** =  significant (α = 0.05).

**Table 3:** Results for the preference test with pro-vitamin A rich and white cassava.

Preference test	Caretakers (n = 30)	Children (n = 30)
Appropriate model	Binomial	Binomial
Max. no. of responses	30	30
No. of responses needed for significance (α = 0.05)	21	21
No. of responses observed favoring yellow cassava	22*	21*
Paired preference µ Test: µ_0_ = 1/2	0.73	0.70

* =  significant (α = 0.05).

Of the 140 caretakers interviewed in the cultural acceptability study, the majority was female (96%), married (87%), between 18 and 50 years of age (83%) having received at least partly primary education (76%). White cassava was cultivated in their farms by 44% of the caretakers and 45% of them reported to have grown it in the previous season. Only 27% reported consumption of white cassava in the past month by their children. Only 15% of the caretakers had heard of pro-vitamin A rich cassava before the interview but none were currently feeding it to their children.

Opinions of the caretakers concerning the different construct are described below per construct.

Construct ‘Behavioral intention’: Almost all caretakers had the intention to prepare pro-vitamin A rich cassava for their children of which 64% were willing to do this two or more times per week.

Construct ‘Health behavior identity’: Forty-seven percent of the respondents reported that eating pro-vitamin A cassava would be good for their child, but 49% were neutral about this.

Construct ‘Perceived barriers 1’: Color, taste, texture and bitterness were worries for 44 to 49% of the caretakers.

Construct ‘Perceived barriers 2’: Most of the caretakers (96%) agreed that cassava peel is poisonous, the crop can easily be destroyed by wild animals (95%), the crop needs a lot of rain to grow (79%) or takes long to mature (71%), it is expensive (60%), not available in the market (56%) and that cuttings (planting material) are not available (61%). Furthermore most caretakers indicated that eating too much raw cassava can upset the stomach (95%) and can cause indigestion (74%).

Construct ‘Cues to action’: Most of the caretakers strongly agreed that information sessions about pro-vitamin A rich cassava (98%), recommendations from health workers (98%) and provision of cassava cuttings (96%) would convince them to prepare pro-vitamin A rich cassava for their children.

Construct ‘External control beliefs’: Caretakers considered themselves to be in control to decide what food to prepare (98%), to buy (99%) for their households and to prepare cassava (99%) for their children.

Construct ‘Subjective norms’: The child itself (76%) and health workers (64%) would mainly influenced the caretakers to prepare pro-vitamin A cassava for their children. Opinions about food that were of importance to the caretakers were those of health workers (99%), the child itself (93%), the father (79%), community members (76%) and village elders (69%).

Construct ‘Knowledge’: Eighty percent of the caretakers did not know that pro-vitamin A rich cassava contains vitamin A and is beneficial for the health of their child. Most agreed with the statement that cassava contains starch (82%). They also indicated that lack of vitamin A is associated with diseases (66%).

Construct ‘Perceived susceptibility’: According to the caretakers, school children in general (81%) and their own school children in particular (75%) were at high risk of developing vitamin A deficiency.

Construct ‘Perceived severity’: Most caretakers agreed that lack of vitamin A made a child susceptible to disease (89%), slowed the growth of the child (87%) and that vitamin A was associated with (night) blindness (69%).

Construct ‘Health value’: Most of the caretakers reported that it was important for them that their child has a good eye sight (99%) and can see during dusk or dawn (99%).

Construct ‘Health behavior identity’: Half of the caretakers (47%) agreed with the statement “Pro-vitamin A rich cassava is good for my child”, and the other half was neutral (49%).

Construct ‘Attitudes towards behavior’: Most of the caretakers indicated that cassava can be prepared in many different ways (98%) and that it is important to give food to their child that can be prepared in different ways (97%). Statements on the properties such as appearance and taste of the cassava were regarded by most of the caretakers as neutral (60–92%). Most of the caretakers indicated that it was important for their child to eat food that is nutritious (99%), sweet (98%), attractive (94%), satisfying (99%), and to eat food that makes the child strong and prevents diseases (99%).

Correlations between the constructs are shown in Figure 1. The constructs ‘Knowledge’, ‘Perceived susceptibility’ and ‘Perceived severity’ were significantly correlated with ‘Health behavior identity’, with the construct ‘Knowledge’ having the highest correlation (rs = 0.354, p<0.01). ‘Health behavior identity’ was significantly correlated with ‘Attitudes towards behavior ( rs = 0.488, p<0.01) and ‘Perceived Barriers 2 (rs = 0.175, P<0.05). ‘Perceived Barriers 1’ was the only construct significantly correlated with ‘Behavioral Intention’ (rs = −0.237, p<0.01) however in an inverse way. Only ‘Cues to action’ (rs = 0.149, p = 0.07) and ‘Control beliefs’ (rs = 0.154, p = 0.078) were borderline significantly correlated with ‘Behavioral intention’.

The relative contribution of the constructs to the three models are shown in Tables 4, 5 and 6. Models were adjusted for interviewer, age and education of the caretaker which increased the variance of the models slightly (referred to as adjΔR^2^. Our constructs explained only a small proportion of the variance in ‘Health behavior identity’ and ‘Behavioral intention’. In the first model the constructs in ‘Background and perception’ explained 23% of the variance (adjΔR^2^ = 9%) in ‘Health behavior identity’, of which ‘Knowledge’ (β = 0.29, P<0.01) was the only significant predictor and ‘Health Value’ was borderline significant (β = −0.14, P = 0.07) (Table 4). The next two models looked at internal (model 2, Table 5) external (model 3, Table 6) predictors of ‘Behavioral intention’. Nine percent of the variance (adjΔR^2^ = 1,9%) was explained by internal factors with ‘Perceived barriers 1’ as the only significant predictor (β = −0.21, P<0.05) and with ‘Health behavior identity’ as borderline significant (β = 0.18, P = 0.07). Eighteen percent of the variance (adjΔR^2^ = 0.05%) was explained by external factors with ‘External control beliefs’ (β = 0.18, P = 0.02) and ‘Cues to action’ (β = 0.51, P<0.01) as significant predictors.

**Table 4:** Predictors of ‘Health behavior identity’ (model 1) to consume pro-vitamin A rich cassava among the study population (n = 140).

Model and related constructs *	Standardized β	P	R^2^	Adjusted R^2^
Model 1 (Y = Health behavior identity)			0.30	0.23
Knowledge	0.29	<0.01		
Perceived susceptibility	0.43	0.63		
Perceived severity	−0.01	0.96		
Health value	−0.14	0.07		

* All models were adjusted for interviewer, age and education of the caretaker.

**Table 5:** Predictors of ‘Behavioral intention’ (model 2) to consume pro-vitamin A rich cassava among the study population (n = 140).

Model and related constructs *	Standardized β	P	R^2^	Adjusted R^2^
**Model 2** (Y = Behavioral intention)			0.17	0.09
Health behavior Identity	0.18	0.07		
Attitudes towards behavior	0.16	0.15		
Perceived barriers – 1	−0.21	0.02		
Perceived barriers – 2	−0.05	0.55		

* All models were adjusted for interviewer, age and education of the caretaker.

**Table 6:** Predictors of ‘Behavioral intention’ (model 3) to consume pro-vitamin A rich cassava among the study population (n = 140).

Model and related constructs *	Standardized β	P	R^2^	Adjusted R^2^
**Model 3** (Y = Behavioral intention)			0.25	0.18
Subjective norm	−0.09	0.32		
External control beliefs	0.18	0.02		
Cues to action	0.51	<0.01		

* All models were adjusted for interviewer, age and education of the caretaker.

## Discussion

We found that both caretakers and children were able to blindly taste a difference between the two types of cassava. When unblinded, they preferred the pro-vitamin A rich cassava over the white cassava, indicating that pro-vitamin A cassava is sensory acceptable. Moreover, almost all caretakers had the intention to prepare pro-vitamin A rich cassava for their children; this intention was most strongly associated with ‘Perceived barriers 1’, ‘Knowledge’, ‘External control beliefs’ and ‘Cues to Action’.

This study has some limitations. Firstly, caretakers for the sensory study were selected by the headmaster of the primary school based on willingness, role in the school and availability. This purposive selection may have resulted in inclusion of caretakers with a certain liking for cassava, or who were instructed as such and this might have contributed to a preference of pro-vitamin A rich cassava. Secondly, the combined model of HBM and TBP has been previously used by others to assess factors that contribute to the consumption of a certain food [12], [13], [14]. It is assumed that intention leads to behavior, however in our study this assumption could not be confirmed as the behavior of eating pro-vitamin A rich cassava does not exist in our study population. To our knowledge, this is the first time the model is used for a biofortified food not yet available in the community. Thirdly, in the cultural acceptability study for the constructs ‘Knowledge’ and ‘Perceived severity’ the answer category “don’t know” was coded as neutral. We assumed that caretakers would have answered ‘neutral’ if they would have had knowledge on the issue asked for. However, this assumption could not be verified and might have influenced the associations of especially ‘Knowledge’ (46% of caregivers replying ‘don’t know’) and ‘Perceived severity’ (19.7% replying ‘don’t know') with health behavior identity. Furthermore the mean construct scores were generally high and correlations between constructs and the variance explained by the models was low. Caretakers may have found it difficult to disagree with the statements. A strong intention to perform a certain behavior can lead to lower correlations between constructs and low variance in the sample population as compared to the general population [27]. We anticipated this and used a 5-point Likert scale instead of a 3-point Likert scale in order to allow for more options within the agreement statements. Low correlation and low variance does not necessarily weaken the effect and importance of the significant predictors to the dependent variable. Similar studies also showed a low correlation and low variance [12], [13], [14]. This may imply that there are other factors contributing to the unexplained variance by the models that are left unexplained. Including a measure of anticipated effect (beliefs about whether or not feelings of regret or upset will follow from not performing the behavior) may help in reducing part of the unexplained variance [28].

Children in our study were less able to discriminate between the pro-vitamin A rich cassava and the white cassava than caretakers (89 versus 130 correct answers). This may be due to lower memory skills of young children affecting their ability to remember a succession of flavors presented for evaluation in a sensory test [21]. Training of the children on how to conduct the tests were held at the schools to improve their performance. Due to time limitations only 30% of the children were trained, which might have contributed to their lower ability to discriminate.

A general concern with introducing vitamin A biofortified staple foods is the change in sensory characteristics of the crop influencing its acceptability by the target population. This does not seem to be a problem for orange fleshed sweet potato as shown in an effectiveness study in Mozambique in which the crop was widely consumed by the community [29]. A study on the acceptability of pro-vitamin A rich yellow maize in Zimbabwe revealed that the yellow maize is perceived as being inferior to white maize. The reason given for the inferiority was that most people received yellow maize as food aid giving yellow maize a negative perception. Authors also stated that due to the higher concentration of oil, fructose and carotenoids in yellow maize chemical changes can easily produce unacceptable organoleptic properties (bad taste) when poorly handled during transportation [30]. Pro-vitamin A rich cassava has no history with food aid. We found that the color and taste of pro-vitamin A rich cassava are perceived as attractive and sweet and yellow cassava was therefore liked more than white cassava, especially by children. There is also some evidence that the carotenoids protect the cassava against post-harvest deterioration and therefore pro-vitamin A cassava can be stored longer than white cassava [31].

Our study population does not consume cassava on a regular basis, with only 27% of the children having consumed cassava in the previous month and 45% of the caretakers currently cultivating cassava on their farms. Since previous experience of consumers influences perception and beliefs, a population that consumes white cassava on a daily basis will probably have stronger beliefs towards the attributes of pro-vitamin A rich cassava. Populations with a higher and more frequent intake of white cassava might be more reluctant towards pro-vitamin A rich cassava than populations with a low and infrequent intake [32]. In our study, the yellow color of the biofortified cassava was perceived as being attractive, but it may be possible that people in areas where white cassava is frequently consumed do not share this opinion.

‘Knowledge’, ‘Perceived susceptibility’ and ‘Perceived severity’ were all significantly correlated with ‘Health behavior identity’ in our study. However, when combined in one regression model, only ‘Knowledge’ remained a significant predictor of ‘Health behavior identity’. Caretakers know about the risks of VAD and the severity of VAD, and also agree that their children are at high risk for VAD. However they are not aware of the link between consumption of pro-vitamin A rich cassava and reducing VAD. Studies on fonio consumption in Mali [12] and amaranth grain in Kenya [13] also found that ‘Knowledge’ was a strong predictor of ‘Health behavior identity’ and this may be because the target population is not yet aware of the extra nutrition and health benefits of the targeted food. Therefore it is important to create awareness about the relation of consuming pro-vitamin A rich cassava and reducing VAD. Comparable research projects concentrating on yellow maize and orange-fleshed sweet potato, confirm that educational sessions and awareness campaigns are effective in increasing consumer acceptability of biofortified crops [30], [33], [34]. A model often used in behavior change research is the transtheoretical model, also called the Stages of Change Model [35]. This model suggests that health behavior change consists of five distinct stages; pre-contemplation, contemplation, preparation, action, and maintenance. Our population can be considered as being in the pre-contemplation or the contemplation stages; although they are aware of the vitamin A deficiency problem, they do not know that consuming pro-vitamin A rich cassava can contribute to a reduction of VAD. Therefore, they may not feel the need or have commitment to incorporate vitamin A rich cassava in their diet. Providing them with knowledge about the relation between vitamin A intake and pro-vitamin A rich cassava might bring them to the next stage of preparation to consume pro-vitamin A rich cassava.

The internal factor ‘Perceived barriers 1’ (related to worries about bitter taste and color) significantly predicted ‘Behavioral intention’. Pro-vitamin A rich cassava is a new cassava variety and it appears that caretakers worry about the taste and appearance of the new cassava. We found in the sensory study that pro-vitamin A rich cassava had a sweet taste (as opposed to bitter) and the color was attractive. We could speculate that these barriers can be taken away if people can taste and see the pro-vitamin A rich cassava.

‘Control beliefs’ and ‘Cues to action’ were significant predictors of intention in the multivariate analysis. ‘Cues to action’ was also found to be a predictor of consumption of amaranth grains in Kenya [13] and of iron-fortified soy sauce in China [14]. However, in these studies, ‘Control beliefs’ was not a predictor. In our community the caretakers feel that they have control over the decision to prepare pro-vitamin A rich cassava for their children. It is unlikely that a caretaker performs a behavior that is outside their control [36] and therefore our finding suggests that when the caretaker is in control of buying and cooking pro-vitamin A rich cassava, she will have the intention to prepare it for their children. We found that ‘Subjective norm’, reflecting social pressure, is not a predictor of intention to consume pro-vitamin A rich cassava in this population. It might be that social pressure is not an issue in our community regarding food choice, particularly cassava. This was also seen in other studies and it was even suggested to be taken out of the model [14], [37]. It could be that staple foods that are generally consumed are well accepted in the community and therefore normative beliefs regarding consumption are low. It has been shown by some studies focusing on sensitive behavior such as binge drinking [38], smoking [39] and driving violations [40], that subjective norms strongly predicted behavior.

Our goal was to determine predictors of consumption of pro-vitamin A rich cassava in order to be able to plan the introduction of this biofortified cassava in a good and successful way. Overall, we found that the yellow color of pro-vitamin a rich cassava is no barrier for consumption in our research population. We found that almost all caretakers had the intention to prepare pro-vitamin A rich cassava for their children and that this intention can be increased by: 1) reducing barriers like worries about color, taste, texture and bitterness; 2) increasing knowledge on vitamin A deficiency and pro-vitamin A rich cassava; 3) empowering mothers to make the decisions for the household on what to cook; 4) involving health workers in the promotion of consumption of pro-vitamin A rich cassava through information sessions about pro-vitamin A rich cassava for caretakers. These initial findings are encouraging, since it underlines the potential of this biofortification strategy to become successful. Similar studies in other populations should reveal whether our findings can be extrapolated to a wider scale.
